# Revision arthrodesis of the ankle: A 4 cannulated screw compression fixation technique

**DOI:** 10.3109/17453674.2011.570676

**Published:** 2011-04-05

**Authors:** Sven Tulner, Mark Klinkenbijl, Gerardus Albers

**Affiliations:** Department of Orthopaedic Surgery, Tergooi Hospitals, Hilversum, the Netherlands

We present a 4 cannulated screw compression technique with use of additional cerclage in 4 patients with nonunion after primary ankle arthrodesis.

## Technique

We performed revision ankle arthrodesis through a bilateral approach with both a lateral and medial incision under tourniquet inflation. First, a transverse fibular osteotomy was performed proximal to the tibiotalar joint through a lateral incision, followed by exposure of the tibiotalar joint. Secondly, a medial incision was made to expose the medial side of the tibiotalar joint. All fibrous tissue was removed from the tibiotalar joint on both sides and the tibiotalar contact area was filled with iliac cancellous bone grafts. Internal fixation was performed with use of large-diameter cannulated screws: one from the posterolateral aspect of the tibia to the talar head, a second from the medial aspect of the distal part of the tibia to the talar dome, a third transverse through the fibula and tibial plafond, and a fourth transverse through the talar dome. To provide axial compression, a cerclage wire was drawn through the 2 transverse cannulated screws and strongly firmed medially and laterally. The tibiotalar joint was positioned in neutral flexion, and slightly posteriorized, the second metatarsal was aligned with the tibial crest, and the hindfoot was positioned in slight valgus. All procedures were performed with use of fluoroscopic guidance to confirm clinical alignment and hardware position (Figure A).

**Figure:**
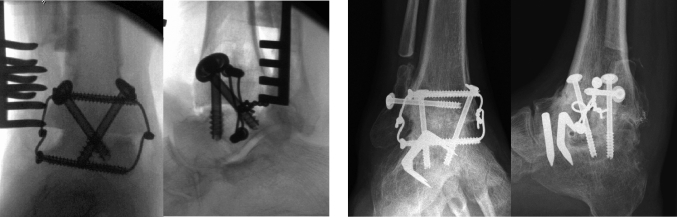
A. Fluoroscopic perioperative images of the right ankle in a patient after revision ankle arthrodesis by use of a 4 cannulated screw fixation technique. The ankle arthrodesis healed after 10 weeks. B. Radiographs 9 months after surgery in a patient with congenital clubfoot deformity previously treated by attempted ankle arthrodesis and subtalar arthrodesis with union.

After-care consisted of non-weight bearing for 4 weeks in a plaster cast. Thereafter, weight bearing in a plaster cast was allowed for 4 additional weeks or longer depending on radiographic union.

## Patients

We used this technique in 4 male patients with nonunion after primary tibiotalar arthrodesis. The mean age was 56 (31–73) years. The indication for initial attempted arthrodesis was posttraumatic osteoarthritis in 2 patients and degenerative osteoarthritis in 2 patients with an underlying neurological disorder (post-poliomyelitis foot deformity in one and congenital clubfoot in the other).

The mean duration of follow-up after ankle revision arthrodesis was 9 (6–12) months. Fusion occurred in all patients after 10–12 weeks (Figure B). No complications were seen, except in 1 patient who had delayed wound healing.

## Discussion

Nonunion after primary ankle arthrodesis is common after attempted arthrodesis. The rate has been reported to range from 0% to 40% (Frey et al. 1994, Coester et al. 2001, Haddad et al. 2007). Different surgical techniques have been introduced to achieve a higher success rate in revision surgery. Generally, current recommendations for primary and revision arthrodesis favor internal compression techniques involving the use of screws or plate fixation or ring external fixation in patients with poor bone stock (Anderson et al. 1997, Easly et al. 2008, Zwipp et al. 2010).

Kirkpatrick et al. (1991) stated that the long tibial moment arm in arthrodesis together with rotational and shear forces of the ankle makes a stable construction difficult. Cheng et al. (2003) advocated fixation with compression by either plating or screw fixation with well-prepared cancellous bone contact surface with good apposition as the key to achieving union. Plaass et al. (2009) used a stable and rigid double anterior plating method for salvage of nonunion after primary tibiotalar arthrodesis with good union rates. It seems that internal fixation with more rigid constructions will provide more stability and shorter union time. Our technique using 2 additional transverse screws and additional cerclage led to rapid union in all 4 patients (Table). A disadvantage of our technique is the need for a bilateral approach. We feel, however, that our 4 cannulated screw compression technique provides more axial tibiotalar compression and therefore may favor earlier union than most previously described internal fixation techniques. The transverse screws offer additional initial stability and compression.

**Table:** Results of revision ankle arthrodesis using internal fixation methods

A	B	C	D	E	F	G	H	I
Kirkpatrick	Variable	Variable **^b^**	10	8	9	20 (unknown)	3	1
Easly **^a^**	Anterolateral	Cancellous (crossed) screws	11	11	8	unknown	4	1
Plaass	Anterior	Double plating	4	4	4	14 (8–22)	no data	
Cheng **^a^**	Anterior	Crossed screws	9	none	8	25 (17–43)	no data	
Anderson **^a^**	Lateral	Crossed screws	13	13	10	25 (9–74)	13	3
Levine	Previous	Crossed screws	11	4	10	15 (6–48)	5	
Verhulst **^a^**	Posterior	Parallel screws	3	3	3	14 (11–17)	0	
Our study	Bilateral	New fixation method	4	4	4	11 (10–12)	1	

**^a^** Only results were used from the patients in this article with tibiotalar pseudarthrosis after primary ankle arthrodesis by internal fixation. Fixation methods are restricted to tibiotalar arthrodesis.

**^b^** Variable: medial compression plate, tibiotalar cancellous screw or tibiotalar crossed screws. A Author B Approach C Fixation method D No. of patients E Bone graft F No. of patients with fusion G Mean time to union in weeks (range) H Complications I No. of patients with amputation
